# Smooth muscle tumor of the placenta - an entrapped maternal leiomyoma: a case report

**DOI:** 10.4076/1752-1947-3-7302

**Published:** 2009-06-17

**Authors:** Katja Murtoniemi, Elina Pirinen, Marketta Kähkönen, Nonna Heiskanen, Seppo Heinonen

**Affiliations:** 1 Department of Obstetrics and Gynecology, Turku University Hospital, P.O.B. 52, FIN-20521 Turku, Finland; 2 Department of Pathology, Kuopio University Hospital, P.O.B. 1777, FIN-70211 Kuopio, Finland; 3 Laboratory of Clinical Genetics, Centre for Laboratory Medicine, Tampere University Hospital, P.O.B. 2000, FIN-33521 Tampere, Finland; 4 Department of Obstetrics and Gynecology, Kuopio University Hospital, P.O.B. 1777, FIN-70211 Kuopio, Finland

## Abstract

**Introduction:**

Neoplasms of the placenta are uncommon. Tumors arising from the placental tissue include two distinct histological types: the benign vascular tumor, chorangioma, and very rarely, choriocarcinoma. Benign leiomyomas, in contrast, are very common tumors of the uterine wall and occur in 0.1% to 12.5% of all pregnant women. However, the incorporation of uterine leiomyoma into the placenta is exceptional and raises the question of its origin. This case is possibly the first report on this kind of a placental tumor which has been examined using both immunohistochemistry and chromosome analysis.

**Case presentation:**

A 34-year-old G4P3 Caucasian woman was followed up antenatally because of a stillbirth in her previous pregnancy. At 36 weeks' gestation, a hypoechoic, 3.6 × 4.2 cm rounded mass was noted within the placenta on ultrasound examination. Histologically, the tumor was a benign leiomyoma and this finding was supported by immunohistochemistry. The newborn infant was male. Chromosomes of the neoplasm were studied by the fluorescence in situ hybridization technique and the tumor was found to carry XX chromosomes.

**Conclusion:**

A rare benign smooth muscle neoplasm involving the placental parenchyma is presented. The tumor was a uterine leiomyoma of maternal origin, which had become entrapped by the placenta. This case report is of interest to the clinical specialty of obstetrics and gynecology and will advance our knowledge of the etiology of placental tumors.

## Introduction

Neoplasms of the placenta are uncommon. Tumors arising from the placental tissue include two distinct histological types: (1) chorangioma, a benign vascular tumor; and (2) choriocarcinoma, which is exceedingly rare. Benign leiomyomas, in contrast, are very common tumors of the uterine wall and occur in 0.1% to 12.5% of all pregnant women [1]. However, the incorporation of a uterine leiomyoma into the placenta is exceptional and raises the question of its origin; whether the tumor is a primary placental or uterine neoplasm. A patient with a placental smooth muscle tumor that appears to be a uterine leiomyoma entrapped entirely in the placenta during its growth is presented.

## Case presentation

A 34-year-old G4P3 Caucasian woman was followed up antenatally because of a stillbirth in her previous pregnancy. She had had mild pre-eclampsia in her first pregnancy and a Caesarean section was carried out after unsuccessful induction of labor. Her second pregnancy and delivery were uneventful. The reason for the stillbirth in her third pregnancy was found to be an umbilical cord knot.

In the pregnancy reported here, our patient had polymorphic eruption of pregnancy (PEP) from 26 weeks' gestation and had three separate courses of oral steroids. Anti-D-antibodies were also found to be increased but in quantitative analyses their concentration, however, remained low. She was hospitalized once for a short period in late pregnancy because of an abnormal fetal heart rate recording.

**Figure 1:**
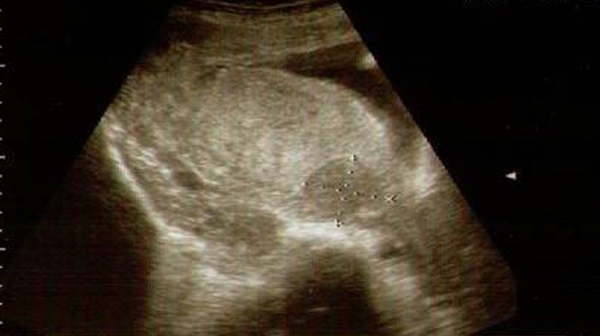
**A hypoechoic, 3**.6 × 4.2 cm rounded mass within the placenta on ultrasound examination at 36 weeks' gestation.

At 36 weeks' gestation, a hypoechoic, 3.6 × 4.2 cm rounded mass was noted within the placenta on ultrasound examination (Figure 1). An ultrasound scan with umbilical artery Doppler measurement after 28 weeks' gestation was normal and it is likely that the leiomyoma had gone unnoticed probably because no special attention had been paid to the placenta on that occasion. Another explanation could be that the leiomyoma grew very rapidly in the third trimester of pregnancy and it was too small to draw appropriate attention in earlier scans.

She had induction of labor due to worsening of PEP at 38 weeks' gestation. A viable male infant weighing 3330 g was delivered with Apgar scores of 9 at one minute and 9 at five minutes. The placenta was removed without difficulty.

**Figure 2:**
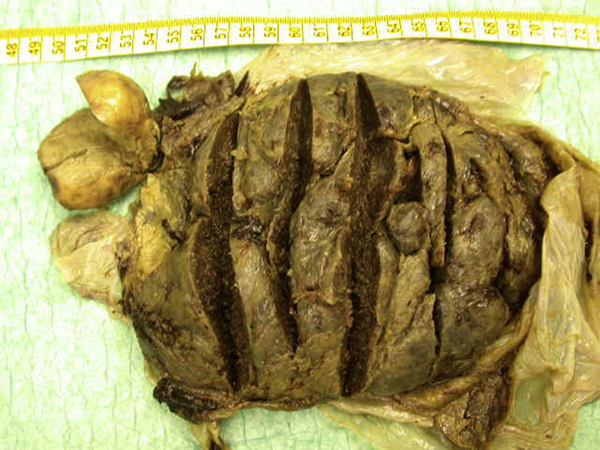
**A round-shaped nodule on the maternal surface of the otherwise normal placenta**. The tumor has been separated from its original site in the placenta.

A round-shaped nodule was noted on the maternal surface of the otherwise normal placenta. The size of the nodule was 4 × 4 × 3 cm. It had a pale cut surface without hemorrhage, necrosis or calcification (Figure 2).

**Figure 3:**
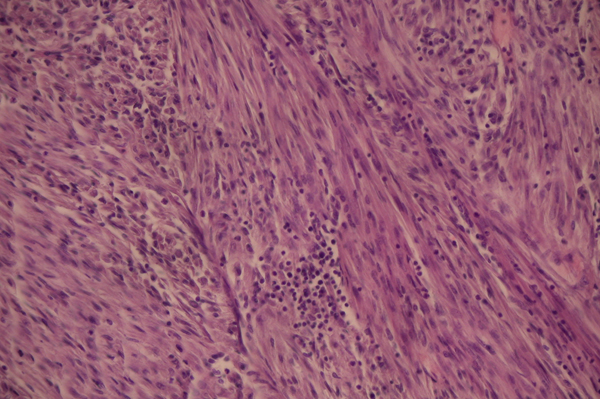
**Histopathological findings are typical for normal leiomyoma**. No atypical features or mitotic activity are seen. Magnification ×20.

Histologically, the nodule was composed of bundles of smooth muscle cells. Nuclei were round or oval shaped and there were no atypical features or mitotic activity (Figure 3). No attached myometrium was identified.

Immunohistochemistry was positive for the smooth muscle actin antigen (Neomarkers, 1:500). Factor VIII related antigen (Dako, 1:750) and CD34 (Becton-Dickinson 1:20) marked only the endothelial cells, whereas cytokeratin of low molecular weight (Becton-Dickinson, 1:20), placental alkaline phosphatase (Dako 1:20) and desmin (Biogenex, 1:50) were negative. The method used for labeling was streptavidin-biotin. The tumor cells were positive for progesterone receptors but negative for estrogen receptors which is typical for leiomyomas during pregnancy [2].

Chromosomes of the tumor were studied from paraffin sections by the fluorescence in situ hybridization technique with X- and Y-chromosome-specific probes and the tumor was found to carry XX chromosomes.

## Discussion

Primary neoplasms of the placenta may originate from trophoblastic or mesenchymal tissues. The latter includes the most common benign vascular tumor of the placenta, the chorangioma. In the literature, three reports of malignant chorangiocarcinoma of the placenta have been published so far, but it has been suggested that chorangiomas with trophoblastic proliferation are more common than suggested by the rarity of reported cases [3]. Neoplasms of the placenta, other than trophoblastic tumors and chorangiomas, are rare and predominantly include teratomas and metastatic maternal tumors [4,5]. In the literature, only seven cases of hepatocellular adenomas have been reported; this is a very rare entity which has been deemed as a benign condition of uncertain histogenesis. A typical lesion, which is described as a solitary and tumor-like nodule involving the placental parenchyma, is composed of fetal-type liver tissue. There are also a few reports of metastasis of smooth muscle tumors arising from sites distant from the placenta [6].

However, the tumor reported in this study did not show any features of malignancy. Primary non-vascular benign mesenchymal tumors are extremely rare; all three reported cases to date have been leiomyomas [7]-[9]. Two of these were primary parenchymal leiomyomas and one was a leiomyoma of the fetal membranes. Furthermore, there is one report of an intraplacental smooth muscle tumor with uncertain malignant potential [10].

Leiomyomas are the most common tumors in women and are found in at least 25% of women in active reproductive life. Leiomyomas are known to be estrogen responsive and may undergo a rapid increase in size during pregnancy. Three theories about the origin of placental leiomyomas have been introduced. First, it may arise from feto-placental mesenchymal tissue. Second, it may represent an intra-endometrial leiomyoma. Third, it may have been a pedunculated uterine leiomyoma incorporated into the placenta during its development and growth.

Abdominal or transvaginal high resolution color Doppler ultrasound has recently made it possible to identify the waveforms obtained from intrauterine masses such as uterine leiomyomas. Knowledge of the characteristics of the expected fetal or maternal waveforms is important to differentiate placental tumors from other intrauterine abnormalities. For instance, in cases of undetermined intrauterine tumors, demonstration of the fetal waveforms strongly suggests a chorangioma, while maternal waveforms suggest a uterine myoma [11].

It is interesting that the situation presented in this report is so rare although leiomyomas are known to be very common in fertile years. One explanation could be that submucosal leiomyomas are often associated with impaired fertility due to implantation failure.

In our patient, the tumor was not entirely inside the placental parenchyma. In line with other reported cases, the tumor was easily separated from the decidua, which has been thought to support a putative origin from feto-placental tissues. Because the newborn was male, it was possible to study the chromosomes of the tumor to find out whether it was of maternal or feto-placental origin. As in the case of Ernst [9], the tumor reported in this study was obviously of maternal origin.

## Conclusion

The tumor was a uterine leiomyoma of maternal origin which became entrapped in the placenta and finally lost its attachment to the uterine wall entirely during pregnancy. Uterine leiomyomas can influence the course of pregnancy [1]. In our patient, there were a number of clinical problems during pregnancy. However, the role of the tumor in jeopardizing the course of pregnancy in this patient is unclear. This is a case report of interest to the clinical specialty of obstetrics and gynecology and it will advance our knowledge of the etiology of placental tumors.

## Abbreviation

PEP: polymorphic eruption of pregnancy.

## Consent

Written informed consent was obtained from the patient for publication of this case report and any accompanying images. A copy of the written consent is available for review by the Editor-in-Chief of this journal.

## Competing interests

The authors declare that they have no competing interests.

## Authors' contributions

KM, NH and EP were the major contributors in study concept and design and writing the manuscript. SH has been involved in drafting the manuscript and revising it critically for important intellectual content. NH was responsible for the care of the patient. EP performed the histological examination of the placenta. MK has made substantial contributions to data analysis and performed the chromosome studies of the tumor. All authors read and approved the final manuscript.
